# Primary pericardial angiosarcoma: case report and review of treatment options

**DOI:** 10.3332/ecancer.2020.1056

**Published:** 2020-06-15

**Authors:** Udit Yadav, Ankit Mangla

**Affiliations:** 1 Department of Medicine, Division of Hematology and Oncology, John H Stroger, Jr Hospital of Cook County, Chicago, IL, USA; 2 Department of Hematology and Oncology, Case Western University School of Medicine, Cleveland, OH, USA

**Keywords:** cardiac tumour, angiosarcoma

## Abstract

A primary cardiac angiosarcoma is a rare type of soft-tissue sarcoma with a high mortality rate. This report describes a young woman who presented with chest pain and worsening shortness of breath over the course of a year. She was diagnosed with and treated for latent tuberculosis and autoimmune pericarditis over the last year, however, her condition kept worsening. Further workup revealed a large pericardial and right atrial mass associated with multiple lung nodules. The biopsy from the lung mass showed angiosarcoma, and she was diagnosed with primary metastatic angiosarcoma of the pericardium. She was treated with doxorubicin and Ifosfamide (AIM-75 regimen), which led to a partial response. However, soon after completion of six cycles, the tumour progressed rapidly, leading to cardio-respiratory failure. In this report, we will discuss the clinical challenges and treatment options (surgical and medical) that are available for treating patients with angiosarcoma of the heart.

## Introduction

Primary cardiac tumours (PCT) are rare tumours, with an autopsy prevalence estimated at 0.001% to 0.03% [1]. A metastatic deposit to the heart from a distant primary is 20–30 times more common than a primary tumour of the heart [2]. A quarter of all PCT are malignant, and 50%–75% of all malignant PCT are angiosarcomas [1, 3–5]. Surgical resection of the tumour is the treatment of choice; however, achieving a microscopically negative surgical margin (R0 resection) is a challenge due to the complex anatomy and proximity with critical structures [6, 7]. Currently, there is no established role of neoadjuvant and adjuvant chemotherapy in patients with localised PCT. Patients who present with metastatic disease have a dismal prognosis with a median life span of less than 6 months [8]. Identifying patients with PCT is a clinical challenge, and imaging and histopathology are critical to establishing a diagnosis. In this report, we will discuss the clinical challenges in a patient with metastatic primary cardiac angiosarcoma. We will also review the literature regarding the treatment of this rare yet fatal cancer.

## Case presentation

A 44-year-old woman presented to the emergency room at our institution with chest pain and shortness of breath. She had no significant past medical history or family history. She denied any history of smoking, alcohol consumption or illicit drug use. A year prior to this visit, she had presented to another hospital with similar complaints. The workup at that time demonstrated pericardial effusion, and the cytology of the pericardial fluid was suggestive of exudative effusion. At that visit, she was diagnosed with latent tuberculosis after a positive tuberculin skin test and received treatment with isoniazid for 6 months. However, her symptoms kept worsening over 6 months, and she presented to our hospital with severe shortness of breath. She gave a history of dyspnea on exertion, occasional squeezing non-radiating chest pain and palpitations. The review of systems was notable for anorexia and significant weight loss over the last six months. At the time of the presentation, she was in significant respiratory distress. The systemic exam was significant for jugular venous distention, bilateral pitting edema of lower extremities, pallor, and use of accessory muscles of respiration. The cardiovascular exam was significant for soft first heart sound and non-palpable apex. The rest of the systemic examination was unremarkable.

**Figure 1.**
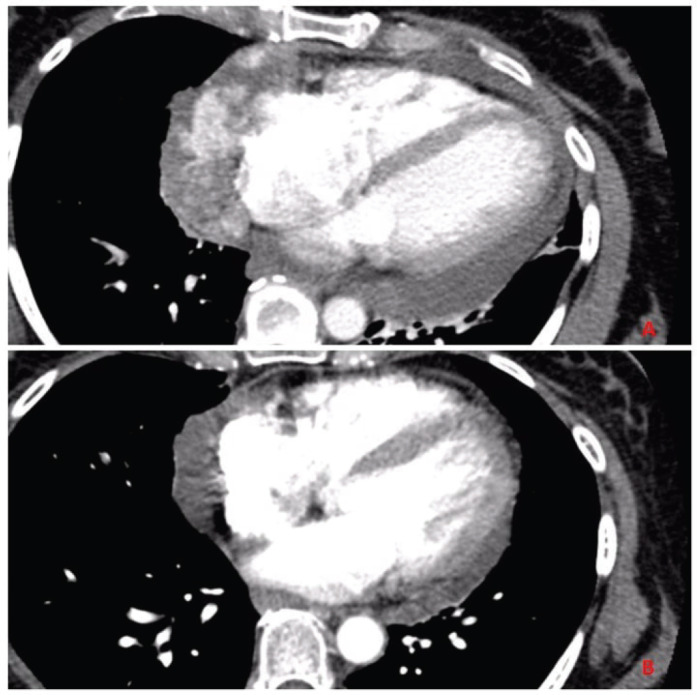
Panel A: CT chest with contrast before initiating chemotherapy shows invasive mass diffusely involving the pericardium and lateral right atrial wall. Panel B: CT chest with contrast after completion of chemotherapy showing partial response in the primary tumour.

**Figure 2.**
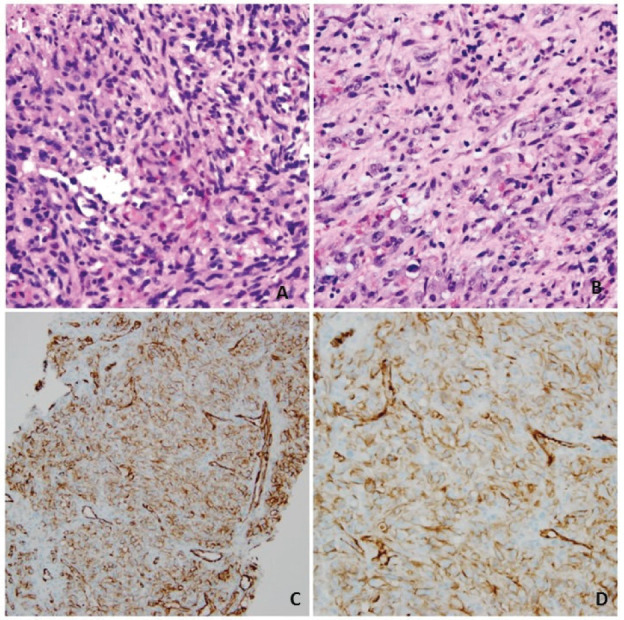
Panel A and B: hematoxylin-eosin stain, original magnifications ×20 and ×40 demonstrating spindle and epithelioid cells with prominent nucleoli, lining anastomosing, and irregular vascular channels. Panel C and D: Immunohistochemical stain for CD31 (PECAM-1), magnifications ×20 and ×40 showing robust staining of malignant cells lining the atypical vascular channels.

Labs at presentation were remarkable for normochromic normocytic anaemia (Hb-11.4 gm/dL) only. The comprehensive metabolic panel, troponin-I and B-natriuretic peptide were normal at presentation. The bedside echocardiogram revealed an ejection fraction of 55%–60%, normal valvular structures, and a moderate, free-flowing, concentric pericardial effusion without tamponade physiology. CT scan of the chest revealed pericardial effusion with a possible pericardial sac mass and multiple bilateral hemorrhagic pulmonary nodules. Cardiac CT showed a highly vascular, right atrial wall soft tissue mass involving the adjacent pericardium associated with large pericardial effusion (Figure 1). CT guided biopsy of the mass showed a tumour with spindle and epithelioid cells, prominent nucleoli and irregular vascular channels (Figure 2). CD31 and CD34 stains were positive in the tumour cells and highlighted the vasculature. The tumour cells were negative for keratin AE1/AE3, EMA, BerEP4, CK7, TTF-1, calretinin, CK5/6, MART-1, HMB45 and S-100.

The patient had metastatic disease at the time of diagnosis. She was treated with AIM 75/10,000 regimen (doxorubicin 25 mg/m^2^ on day 1–3, ifosfamide 2,000 mg/m^2^ on day 1–5 with mesna and granulocyte colony-stimulating factor support). She completed six cycles of treatment with 25% dose reduction and minor treatment delays due to two episodes neutropenic fever. A partial response was noted after six cycles with at least 50% reduction in the tumour burden. However, within 6 weeks of completing treatment, she was diagnosed with progressive disease and opted for hospice.

## Discussion

Primary cardiac sarcomas are rare and pose a diagnostic challenge to the clinician. The symptoms are varied where patients can present with congestive heart failure secondary to intracardiac obstruction, rhythm disturbances, embolisation of the tumour, chest pain, shortness of breath and/or constitutional symptoms [9]. Left and right heart sarcomas are different in presentations and characteristics. Where the left-sided tumours are more solid, less infiltrative, and tend to metastasize later; right-sided tumours are bulky, infiltrative and tend to metastasize early [10]. Imaging is critical to establish the presence of a mass, defining its mobility (useful in determining prognosis and embolic potential), presence of myocardial invasion, and location of the mass within the cardiac chambers. A three-dimensional echocardiogram (3-D Echo) is preferred to describe the tumour accurately [10]. Cardiac magnetic resonance (MR) is the best imaging technique as it provides precise spatial and contrast resolutions. Positron emission tomography (PET) is useful in establishing metastatic spread [10]. A combination of cardiac MR, 3-D Echo and PET scan is the best way to image patients with primary cardiac angiosarcoma [10]. Soft-tissue sarcoma (STS) is a heterogeneous group of disease and over 70 different molecular subtypes of are defined [11]. A detailed pathologic description and immunohistochemistry staining pattern is a must to establish the exact histologic subtype of sarcoma. Angiosarcoma typically presents as an abnormal, pleomorphic, malignant endothelial cells which express von Willebrand factor, CD34, CD31, ulex europaeus agglutinin one and vascular endothelial growth factor (VEGF) [12].

Clinical management of primary cardiac angiosarcoma involves a multimodality approach, including cardiothoracic surgery, radiation oncology and medical oncology. The median overall survival (OS) for patients with any PCT receiving surgical resection is 12 months compared to 1 month for those who do not receive any treatment [3]. Surgical resection with a microscopically negative margin (R0) is associated with the longest median survival of 17 months, followed by R1 resection (microscopically positive margins only) which is associated with a median survival of 6 months [6–8, 13]. The role of adjuvant radiotherapy (RT) and chemotherapy remain controversial in the treatment of patients with cardiac sarcoma. A review of the SEER database showed that although RT was associated with a longer median OS (11 months for those receiving RT versus 4 months for those who did not receive RT), this was not a statistically significant association, especially when accounted for variability in surgical technique [3]. The authors postulated that RT should not be dismissed as a treatment modality as local control of the tumour seems to be associated with best survival outcomes [3]. The role of adjuvant chemotherapy in patients with primary cardiac sarcoma after resection remains anecdotal [14]. In an observational study of 15 patients with primary cardiac sarcoma (regardless of subtype) undergoing surgical resection, adjuvant chemotherapy resulted in an average OS of 12 months [14]. The patients received either a combination of cyclophosphamide, vincristine and dacarbazine (four patients); Ifosfamide alone (nine patients); methotrexate and vincristine (one patient) or doxorubicin alone in one patient. Even in this study, R0 resection was associated with the best survival outcome (median disease-free survival (DFS) of 18 months and OS of 23.5 months). Those who had margin involvement on the tumour specimen (R1 or R2 resection) had poor DFS (4.5 months) and OS (7 months), indicating that a positive margin weighs heavily in determining the survival of a patient with PCT [13].

Neoadjuvant chemotherapy in patients with any STS is gaining momentum, especially for patients in whom surgical resection is likely to result in R1 or R2 resection [11]. In a study of 27 patients diagnosed with cardiac sarcoma (11 patients had angiosarcoma), perioperative chemotherapy (neoadjuvant, adjuvant, or both) resulted in a survival rate of 80.9% at 1 year and 61.6% at 2 years. (9) The combination of doxorubicin and Ifosfamide or gemcitabine and docetaxel or single-agent high dose ifosfamide was the chemotherapy of choice [9]. In a follow-up study, the same group reported outcomes of 44 patients with right heart sarcoma (30 patients had angiosarcoma). The findings again confirmed the OS benefit of R0 resection over R1 resection (53.5 months versus 9.5 months, respectively). In addition to this, they also reported that adding neoadjuvant chemotherapy improved OS from 9.5 to 20 months [4]. They also reported that patients receiving neoadjuvant chemotherapy have a higher rate of R0 resection rate; however, this observation could not achieve statistical significance. More radical and invasive measures like cardiac autotransplant followed by adjuvant chemotherapy do not result in a better survival outcome [15]. For patients with left heart sarcoma, Methodist hospital (Houston) follows the protocol of cardiac autotransplant with or without pneumonectomy, followed by adjuvant chemotherapy [16]. Despite high 30-day mortality of 57% in the pneumonectomy group and 11% in autotransplant only group, 1-year, and 2-year survival rates are considerably high (58% and 32%, respectively). As of now, cardiac autotransplant for any PCT remains investigational.

The management of metastatic cardiac angiosarcoma relies on systemic chemotherapy. Anthracycline-based regimens are associated with a response rate of 16%–27% and median survival of up to 12 months [12, 17]. Taxanes (both paclitaxel and docetaxel) have shown an exceptional activity in angiosarcomas due to their anti-angiogenic properties [12]. Although the efficacy of anthracycline and taxanes in angiosarcomas have never been compared in a prospective trial, the retrospective data suggests a similar survival benefit with the two drugs. Due to the vascular nature of the tumours, early interest in anti-VEGF therapy led to phase I/II trials with bevacizumab, sunitinib, sorafenib and axitinib. Although bevacizumab showed promising activity with a 12% overall response rate, its addition to chemotherapy could not significantly improve progression-free survival compared to chemotherapy alone [12, 18]. The evidence regarding the efficacy of checkpoint inhibitors in treating advanced sarcomas remains anecdotal [19, 20]. The ALLIANCE trial data with PD-1/PDL-1 and CTLA-4 antibodies demonstrated a response rate of 5%–20% with similar rates of immune-related adverse events, as seen in clinical trials with other tumour types [21, 22].

## Conclusion

In conclusion, primary cardiac angiosarcoma is a rare soft-tissue sarcoma associated with a high mortality rate. The clinical diagnosis is challenging for most clinicians and an accurate diagnosis requires cardiac MR, 3-D echo and the PET scan. Surgical resection with an R0 margin is associated with the best outcome. Although there is a paucity of prospective trials to validate any particular approach in the treatment of cardiac angiosarcoma, there is a benefit of adding chemotherapy and radiation therapy to surgical resection. Anthracycline and taxanes are the most active cytotoxic drugs in this tumour subtype.

## Funding statement

The authors do not have any financial relations to disclose.

## Contribution statement

UY wrote the manuscript. AM revised the manuscript and provided expert opinion.

## Conflicts of interest

The authors do not have any conflicts of interest to declare.

## Patient consent

Patient consent was obtained prior to the preparation of this report.
